# A Toolkit for Monitoring Immunoglobulin G Levels from Dried Blood Spots of Patients with Primary Immunodeficiencies

**DOI:** 10.1007/s10875-023-01464-0

**Published:** 2023-03-21

**Authors:** Hanna Haberstroh, Aleksandra Hirsch, Sigune Goldacker, Norbert Zessack, Klaus Warnatz, Bodo Grimbacher, Ulrich Salzer

**Affiliations:** 1 Institute for Immunodeficiency, Center for Chronic Immunodeficiency (CCI), Medical Center - University of Freiburg, Faculty of Medicine, University of Freiburg, Freiburg, Germany; 2 DZIF - German Center for Infection Research, Satellite Center, Freiburg, Germany; 3 Department of Rheumatology and Clinical Immunology, Medical Center - University of Freiburg, Faculty of Medicine, University of Freiburg, Freiburg, Germany; 4 Center for Chronic Immunodeficiency (CCI), Medical Center - University of Freiburg, Faculty of Medicine, University of Freiburg, Freiburg, Germany; 5 Takeda Pharma Vertrieb GmbH & Co. KG, Berlin, Germany; 6 CIBSS - Centre for Integrative Biological Signalling Studies, Albert-Ludwigs University, Freiburg, Germany; 7 RESIST - Cluster of Excellence 2155 to Hanover Medical School, Satellite Center Freiburg, Freiburg, Germany

**Keywords:** Dried blood spot, immunoglobulin replacement therapy, nephelometry, primary immunodeficiency, remote monitoring, subcutaneous immunoglobulin

## Abstract

**Purpose:**

This study assessed whether measuring immunoglobulin G (IgG) from dried blood spots (DBSs) using nephelometry is a suitable remote monitoring method for patients with primary immunodeficiencies (PID).

**Methods:**

Patients receiving immunoglobulin replacement therapy for PID were included in this non-interventional single-arm study (DRKS-ID: DRKS00020522) conducted in Germany from December 4, 2019, to December 22, 2020. Three blood samples, two capillary DBSs (one mail-transferred and the other direct-transferred to the laboratory), and one intravenous were collected from each patient. IgG levels were determined using nephelometry. IgG levels were summarized descriptively, and significant differences were assessed using Wilcoxon matched-pairs signed-rank tests. Correlation and agreement between IgG levels were assessed using Spearman correlation and Bland–Altman analyses, respectively.

**Results:**

Among 135 included patients, IgG levels measured from DBS samples were lower than those measured in serum (*p* < 0.0001). There was no significant difference between IgG levels in direct- and mail-transferred DBS samples. There was a high degree of correlation between IgG levels in serum samples and DBS samples (*r* = 0.94–0.95). Although there was a bias for higher levels of IgG in serum than in DBS samples, most samples were within the 95% interval of agreement. There was a high degree of correlation between IgG levels measured in direct- and mail-transferred DBS samples (*r* = 0.96) with no bias based on the shipment process and most samples within the 95% interval of agreement.

**Conclusion:**

Monitoring IgG levels from DBS samples is a suitable alternative to the standard method, and results are not substantially affected by mailing DBS cards.

**Supplementary Information:**

The online version contains supplementary material available at 10.1007/s10875-023-01464-0.

## Introduction

Patients with primary immunodeficiencies (PID) are at risk of chronic or recurrent infections; some patients may also have a high susceptibility to non-infectious complications such as autoimmune conditions and malignancy [1, 2]. For patients with PID caused by antibody deficiencies, long-term plasma-derived immunoglobulin replacement therapy (IGRT) represents the standard of care [3, 4]. IGRT can be delivered intravenously at a medical facility or subcutaneously, which gives patients the option to receive infusions at home [5]. While receiving IGRT, patients’ immunoglobulin G (IgG) levels need to be monitored to help clinicians to ensure that patients are receiving a therapeutic dose [6]. However, home-based monitoring of serum IgG levels is not yet readily available owing to the need to collect an intravenous (IV) blood sample by venipuncture using the current standard method [6].

The dried blood spot (DBS) card is a collection device that allows patients the flexibility to collect capillary blood samples themselves at home. To collect a sample the patient performs a finger prick and places one or two drops of capillary blood onto a DBS card, which is then sent to a laboratory for elution and analysis. In the setting of the COVID-19 pandemic, the DBS method has been shown to be a useful remote monitoring tool for the delivery of routine ambulatory care while minimizing hospital visits and the risk of virus exposure [7, 8]. A remote monitoring method such as DBS sampling would be beneficial to monitor IgG levels in patients with PID who are at high risk of infections. Previous research has demonstrated that eluates from DBSs can be used to monitor immunoglobulin levels via multiplexed Luminex assays (Luminex Corporation, Austin, USA) or enzyme-linked immunosorbent assays (ELISA), and the results are comparable to those obtained from serum [9, 10].

In this study, we adapted the analysis method used by Yel et al. [10] to develop a toolkit for monitoring serum IgG levels via nephelometry using DBS cards. Our primary objective was to assess the suitability of this toolkit in determining serum IgG levels in patients with PID [11]. Our secondary objective was to assess the impact of DBS shipment (direct and mail transfer) on the results from the IgG assay.

## Methods

### Preliminary Tests of DBS Card Stability

To assess the stability of DBS cards, DBS samples from three different anonymous healthy controls were collected on Whatman 903 protein saver cards (GE Healthcare Life Sciences, Boston, USA). Cards were stored at − 20 °C, 4 °C, room temperature, and 37 °C for a maximum of two (− 20 °C, 4 °C, and 37 °C) or four (room temperature) weeks. Blood was eluted from DBS cards at 24 h, 3 days, 1 week, 2 weeks, 3 weeks, or 4 weeks after sampling, and IgG levels in the eluates were determined by nephelometry. The elution process is described in the Online Resource in the Supplementary Material.

### Study Design

This was a non-interventional single-arm study (DRKS-ID: DRKS00020522) conducted at one site in Freiburg, Germany. Patients were enrolled in the study from December 4, 2019, to December 22, 2020; the planned target study population size was 200 patients.

### Patients

Patients were included if they met the following inclusion criteria: had a diagnosis of a PID, were receiving IGRT, were aged 18 years or over, had an IV blood sample taken for serum IgG analysis at a regular hospital visit, were able to perform a finger prick (with or without the support of medical staff), and provided voluntary, informed, written consent to participate in the study (Institutional Review Board, University of Freiburg #514/18). During the study, 15 patients who were not receiving IGRT at the time of enrollment into the study were included in a deviation from the protocol. Patients were excluded if they had participated in any other interventional clinical study in the 30 days prior to enrollment.

### Blood Sample Collection

For each patient, an IV blood sample was drawn and two capillary blood samples by finger prick were collected on separate DBS cards during the same visit at the Center for Chronic Immunodeficiency (CCI) outpatient clinic, held at the Medical Center, University of Freiburg, Germany. The sequence of IV and finger prick blood draws was randomized using a pre-determined randomization sequence, and IV and finger prick samples were taken within 15 min of each other in different arms. IV blood samples were transferred directly to the laboratory via internal hospital transport. To collect a blood sample on a DBS card, the selected puncture site (e.g., the side of the middle finger) was cleaned using an alcohol wipe and left to dry for 10 s, and a lancet was used to puncture the site, and a drop of blood was formed by massaging the finger. One or two drops of blood were then placed on an application area of a Whatman 903 protein saver card until the field was covered as completely as possible. The Whatman cards were then allowed to dry at room temperature for at least 4 h. The two DBS cards from each patient were randomized to direct transfer or mail transfer to the laboratory. Mail-transferred samples were shipped in the regular mail at ambient temperatures and were received 1–6 days after dispatch (mean 1.8 days). The mean duration of storage at 4 °C (i.e., the time between the arrival of the shipped sample and analysis) was 8 days. Sample analysis was performed once weekly.

### IgG Assay

Serum was obtained from IV blood by centrifugation; IgG levels were then determined by nephelometry using N antiserum against human IgG (Siemens Healthcare GmbH, Erlangen, Germany) and the settings for serum IgG determination (dilution 1:400) on an Atellica NEPH 630 System (Siemens Healthcare GmbH). Separate standard curves and internal controls were used according to the manufacturer’s instructions. Two replicates from each serum sample were measured to control for pipetting and measurement error.

DBS quality was assessed prior to analysis by eye, and low-quality samples were excluded. Low-quality samples were defined as insufficiently filled or unevenly distributed spots on the card, for which spot extraction was not feasible. DBS cards were kept at 4 °C for a maximum of 10 days before elution. The protocol for determining IgG levels in eluates from DBS samples was adapted from that used by Yel et al. [10] and is described in the Online Resource in the supplementary material, the key difference being the use of nephelometry in the current study. Nephelometry is a sensitive method [11] and has been shown to be compatible with DBS samples in preliminary testing; IgG concentrations determined in eluates from DBS were close to those in serum using the standard method. Settings for analysis of IgG in cerebrospinal fluid (CSF) on the nephelometer were used, because IgG levels in concentrated DBS eluates were expected to be slightly higher than in CSF; this analysis used a predilution factor of four. The reagents used (except the internal controls, dilution on the instrument, and the dedicated suitable calibration curve) are the same for IgG measurement in both serum and CSF. The nephelometric reference controls LC1 and LC2 (Siemens Healthcare, Erlangen, Germany) were used to control for the accuracy and precision of the nephelometric analysis of IgG levels using settings for CSF. These controls were run in parallel with each round of elution once a week.

### Statistical Analysis

IgG levels in serum and in eluates from direct-transferred DBS and mail-transferred DBS were summarized descriptively and compared using Wilcoxon matched-pairs signed-rank tests to test for statistically significant differences (*p* ≤ 0.05) between the three groups. Spearman rank correlation analysis was used to assess correlation, and the Bland–Altman approach [12] was used to assess agreement, between IgG levels from serum and DBS and from direct- and mail-transferred DBS. A non-quantifiable sample was defined as a sample with a concentration below the lower limit of quantification. The lower limit of quantification was reagent- and lot-specific and was approximately 2.5 g/L with the settings for analysis of CSF IgG. GraphPad Prism 9 software (GraphPad Software, San Diego, CA, USA) was used to perform statistical analysis.

## Results

### Preliminary Tests of DBS Card Stability

**Fig. 1:**
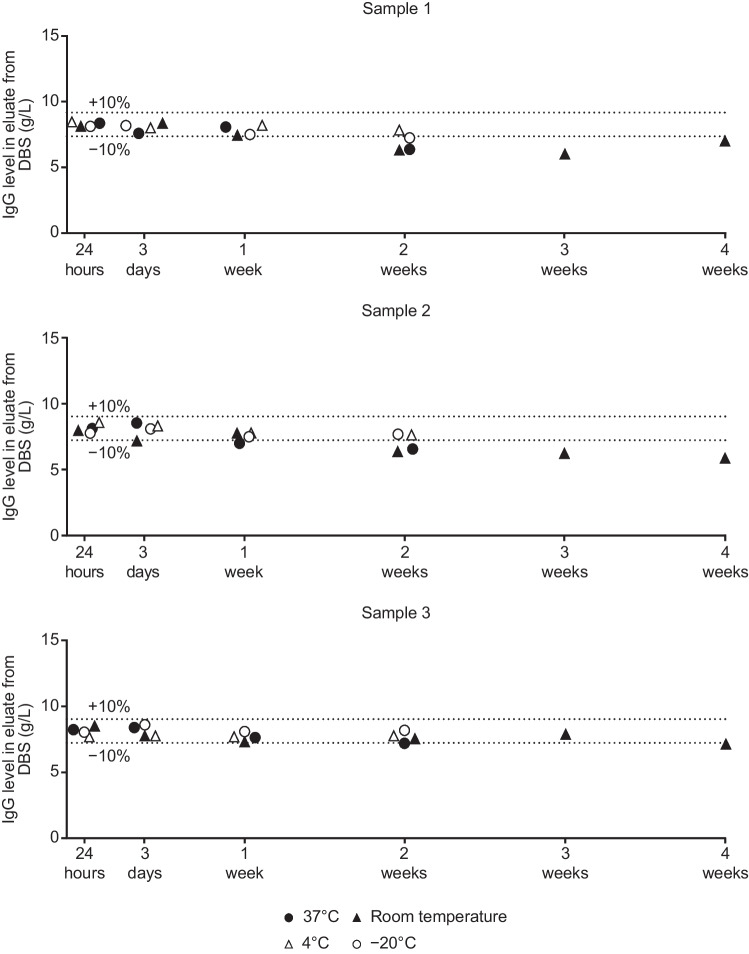
DBS sample stability at different temperatures and durations of storage. The three samples were collected from three different anonymous healthy controls. *DBS*, dried blood spot; *IgG*, immunoglobulin G

The stability of DBS cards stored at different temperatures for up to 4 weeks is shown in Fig. 1. IgG levels remained within a 10% deviation when DBS cards were stored for up to one week at all temperatures. For DBS cards stored at room temperature or 37 °C, greater than 10% deviations were recorded when cards were stored for more than 1 week.

### Patient Disposition and Characteristics

Among the 150 patients enrolled in the study, 15 had incomplete or low-quality samples (DBS spot extraction not possible [*n* = 13], serum sample missing [*n* = 1], or documentation missing [*n* = 1]) and were excluded from analyses. A total of 135 patients with complete data sets were included in the analysis. An ad hoc interim analysis indicated that this population size was sufficient for statistical significance analysis [13]; therefore, it was decided to lower the number of patients enrolled from the planned 200. Patient characteristics are shown in Table 1. Patient ages ranged from 20 to 78 years, and there were approximately equal proportions of male and female patients. Most patients (73.3%) had a diagnosis of common variable immunodeficiency and were receiving IGRT; 15 patients (11.1%) not receiving IGRT were included in the study in a deviation from the inclusion criteria owing to recruitment difficulties associated with the COVID-19 pandemic.

**Table 1:** Patient characteristics

Parameter	All patients (*N* = 135)
Age, years, median (range)	47 (20–78)
Sex, *n* (%)	
Male	67 (49.6)
Female	68 (50.4)
Type of PID, *n* (%)	
CVID	99 (73.3)
sIgAD/sIgGscD	15 (11.1)
Combined immunodeficiency	6 (4.4)
Unclassified immunodeficiency with antibody deficiency	4 (3.0)
Agammaglobulinemia	3 (2.2)
Other^a^	8 (5.9)
Type of IGRT, *n* (%)	
IVIG	42 (31.1)
SCIG	78 (57.8)
None	15 (11.1)
IgG dose per month, g, mean (range)	33.5 (5–90)

^a^Other diagnoses comprised autosomal dominant hyper IgE syndrome (*n* = 2), ADA2 deficiency (*n* = 2), carrier of NEMO gene mutation (*n* = 1), XIAP deficiency (*n* = 1), idiopathic CD4 T-cell deficiency (*n* = 1), and intestinal lymphangiectasia and protein loss (*n* = 1). *ADA2*, adenosine deaminase 2; *CD4*, cluster of differentiation 4; *CVID*, common variable immunodeficiency; *IgG*, immunoglobulin G; *IGRT*, immunoglobulin replacement therapy; *IVIG*, intravenous immunoglobulin; *NEMO*, nuclear factor-kappa B essential modulator; *PID*, primary immunodeficiencies; *SCIG*, subcutaneous immunoglobulin; *sIgAD*, selective immunoglobulin A deficiency; *sIgGscD*, selective immunoglobulin G subclass deficiency; *XIAP*, X-linked inhibitor of apoptosis

### Comparison of IgG Levels in Serum and DBS

Median IgG levels were 9.5 g/L in serum and 8.3 g/L for both direct- and mail-transferred DBS samples. Compared with serum, eluates from direct- and mail-transferred DBS samples contained significantly lower IgG levels (*p* < 0.0001 for both comparisons). There was no significant difference between IgG levels from direct- and mail-transferred DBS samples (*p* = 0.8977).

**Fig. 2:**
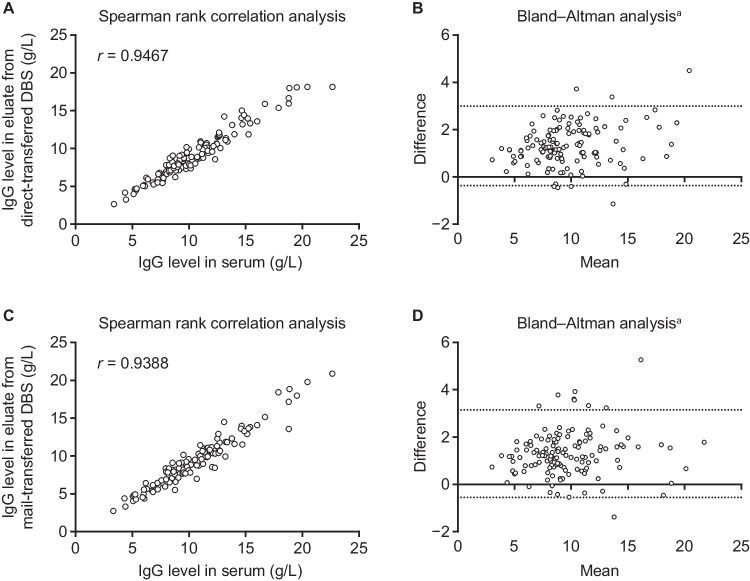
Comparison of IgG levels in serum versus eluate from direct-transferred (**A**, **B**) and mail-transferred (**C**, **D**) DBS samples using spearman rank correlation analysis and Bland–Altman Analysis. ^a^Dotted lines show the 95% limits of agreement. *DBS*, dried blood spot; *IgG*, immunoglobulin G

Spearman rank correlation analysis of paired samples showed a high degree of correlation between IgG levels from serum compared with direct-transferred DBS (*r* = 0.95) (Fig. 2A) and mail-transferred DBS (*r* = 0.94) (Fig. 2C). Bland–Altman analysis showed a bias toward higher levels of IgG in serum than DBS eluates; however, most levels were within the 95% interval of agreement, indicating a good level of agreement between IgG levels from serum compared with eluates from direct-transferred DBS samples (Fig. 2B) and mail-transferred DBS samples (Fig. 2D). A similar strong correlation between DBS samples and serum IgG was also observed in the subgroup of patients who were not receiving IGRT at enrollment into the study (serum versus direct-transferred DBS, *r* = 0.96; serum versus mail-transferred DBS, *r* = 0.99; both *p* < 0.0001). Consistent with the group with IGRT, median IgG levels in serum were also higher than in DBS samples (serum IgG: 8.8 g/L; direct-transferred DBS: 7.9 g/L; mail-transferred DBS: 8.0 g/L).

**Fig. 3:**
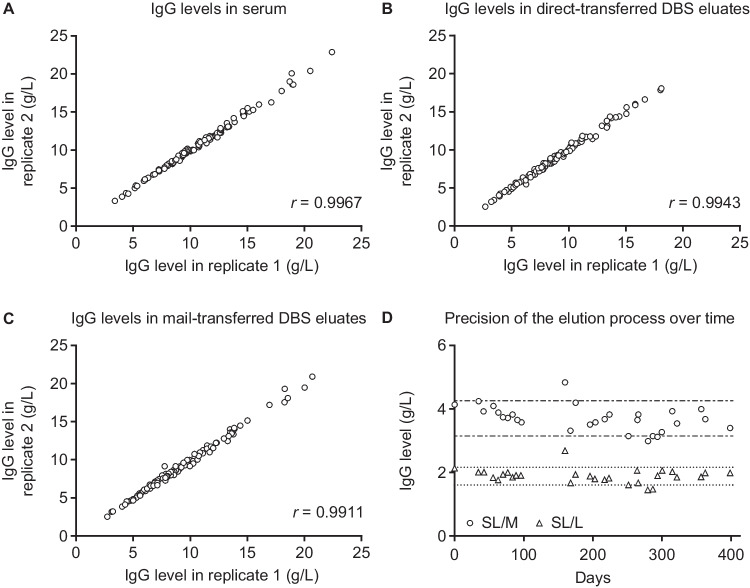
Correlation of replicate measurements using Speakman rank correlation analysis (**A**–**C**) and precision of the elution process over time using controls (**D**). In part D, SL/L and SL/M (Siemens Healthcare, Erlangen, Germany) are two different standard nephelometric reference protein controls. Dashed and dotted lines represent ± 20% of the mean value from the 29 independent SL/M measurements and from the 29 independent SL/L measurements shown in the figure, respectively*. DBS*, dried blood spot; *IgG*, immunoglobulin G

Replicate analysis showed a high degree of correlation among replicate measurements, indicating a low risk of pipetting errors during direct sample preparation and high precision of repetitive nephelometric measurements (Fig. 3A–C). A provisional quality control process showed good-to-moderate precision of the elution process over the course of the study (Fig. 3D).

### Comparison of IgG Levels in Direct- Versus Mail-Transferred DBS

**Fig. 4:**
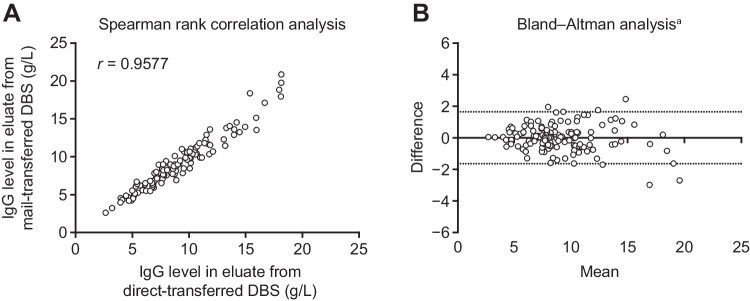
Comparison of IgG levels in eluates from direct- versus mail-transferred DBS samples using Spearman rank correlation analysis (**A**) and Bland–Altman analysis (**B**). ^a^Dotted lines show the 95% limits of agreement. *DBS*, dried blood spot; *IgG*, immunoglobulin G

Spearman rank correlation analysis of paired samples showed a high degree of correlation between IgG levels in eluates from direct-transferred and mail-transferred DBS samples (*r* = 0.96) (Fig. 4A). Bland–Altman analysis showed a good level of agreement between IgG levels from direct- and mail-transferred DBS samples, and most levels were within the 95% interval of agreement, indicating that the shipment process had little impact on IgG levels (Fig. 4B). Analysis of the subgroup of 15 patients who were not receiving IGRT at enrollment showed a similar strong correlation between IgG levels determined from direct- and mail-transferred DBS samples (*r* = 0.98).

## Discussion

Irrespective of the shipment method, IgG levels in DBS eluates showed a high degree of correlation to those measured from serum. There was also a good level of agreement between sample types using Bland–Altman analysis, which is more sensitive to potential bias than Spearman rank correlation analysis. These observations were similar to the correlation between IgG levels from DBS eluates and serum seen by Yel et al. [10], although a study to quantify immunoglobulin isotypes from newborn DBS found correlations for only some subclasses of IgG [9].

In the current study, IgG levels from DBS eluates were consistently lower than those from serum samples. Possible explanations include the DBS sampling procedure or the properties of capillary blood compared with venous blood. Some studies have reported lower levels of total protein (including gamma globulins) in capillary blood than in venous blood [14, 15]. However, results can be variable, and studies that have determined immunoglobulin levels from DBS using different immunoassays (i.e., Luminex assay or ELISA) have reported similar, higher, or lower levels of immunoglobulin isotypes in DBS than in serum/plasma [9, 10]. Establishing reference ranges specific to IgG levels determined from capillary blood in DBS and appropriate reference and control materials for standardization will help to adjust for this.

Our findings indicate that nephelometry is sensitive enough to quantify IgG levels in DBS eluates. This was achieved by using the assay settings for CSF analysis, which were already preinstalled on the nephelometric analyzer. The system used does not have an open platform, meaning that tailored, customized assays cannot be programmed by users. If the program and controls were tailored to DBS eluates, it may be possible to produce more accurate measurements in the future. Nephelometry is already the gold standard for determining IgG levels in serum, and nephelometric analyzers and standard processes are in place at many medical facilities. Furthermore, nephelometry requires a lower working dilution of DBS eluates (1:4) than ELISA (≥ 1:500 000) [10], which reduces the number of dilution steps, thus the risk of inaccurate results. Nephelometry is therefore a suitable option for monitoring the IgG levels in patients with PID to ensure that they do not fall below the therapeutic dosing level of IGRT.

IgG levels from DBS samples were not significantly affected by the shipment method, with a high degree of correlation and a good level of agreement between results from direct-transferred and mail-transferred samples. This is similar to the findings of Prinsenberg et al., who used DBS cards to collect samples to monitor viral RNA levels of patients with the human immunodeficiency and hepatitis C viruses [16], indicating that even sampling of sensitive RNA is possible using DBS cards. The preliminary temperature stability analyses also indicate that the DBS samples produce reliable results when stored for up to 1 week, similar to findings reported by Yel et al. [10]. Taken together, our results suggest that samples can be shipped during all seasons at temperatures ranging from − 20 to 37 °C, assuming regular mailing turnaround times.

The DBS sampling method used herein would have been of benefit to patients during the early stages of the COVID-19 pandemic. Our data suggest that IgG levels from DBS samples remain stable when mail-transferred to the laboratory, allowing patients to monitor their IgG levels while avoiding the infection risk associated with healthcare settings [17]. The DBS sampling method would also allow immunoglobulin level monitoring where no point-of-care testing is available, such as in rural areas. This IgG-DBS toolkit could also be applied to screening and diagnosis. However, ensuring the quality of the sample is a likely challenge if in-person patient training on sample collection is not feasible. Diagnosis of PID or hypogammaglobulinemia would also require the determination of IgA and IgM levels, which were not tested in the current study. Further work is required to determine whether these immunoglobulin classes can be measured with sufficient accuracy and sensitivity using nephelometry.

A limitation of the DBS sampling method is that the volume of blood from DBS cards cannot be calculated exactly because of the hematocrit effect on blood viscosity (i.e., different hematocrit levels in blood result in DBS cards containing different amounts of blood) [18, 19]. Various approaches have been proposed to correct for the hematocrit effect; these include using an entire DBS with a precise volume of blood applied, using dried plasma spots, and adapting the design of DBS collection to minimize the hematocrit effect [18, 19]. A limitation of the study was that it was not possible to fulfill the inclusion criterion that all patients were to have received IGRT prior to enrollment in the study; this was due, in part, to the first months of the COVID-19 pandemic, during which time patients with immunodeficiencies, regardless of whether receiving IGRT or not, were discouraged from attending routine clinic visits, thus affecting enrollment. Another limitation of the study is that longitudinal analysis of the same patients could not be performed.

## Conclusions

The current study establishes that home-based monitoring of IgG levels via DBS is a suitable and patient-friendly alternative to the standard method of using IV-drawn blood and that the results are not affected substantially by mail transfer of the DBS cards to the laboratory. We also demonstrate that nephelometry is a robust method to determine IgG levels in DBS samples. The IgG-DBS toolkit described has the potential to be applied in routine clinical practice.

## Supplementary Information

Below is the link to the electronic supplementary material.

Supplementary file1 (DOCX 21 KB)

## Data Availability

The data sets generated during and/or analyzed during the current study are available from the corresponding authors upon reasonable request.
