# Supplementary material for: A Toolkit for Monitoring Immunoglobulin G Levels from Dried Blood Spots of Patients with Primary Immunodeficiencies
Source: J Clin Immunol. 2023 Mar 21;43(6):1185–92. doi: 10.1007/s10875-023-01464-0 (PMC10027597; doi:10.1007/s10875-023-01464-0)
Supplement: Supplementary file 1 — Supplementary file1 (DOCX 21 KB) [file 10875_2023_1464_MOESM1_ESM.docx]

# Supplementary Information

**Manuscript title:**

A Toolkit for Monitoring Immunoglobulin G Levels from Dried Blood Spots of Patients with Primary Immunodeficiencies

**Journal:**

Journal of Clinical Immunology

**Authors:**

Hanna Haberstroh, Aleksandra Hirsch, Sigune Goldacker, Norbert Zessack, Klaus Warnatz, Bodo Grimbacher, Ulrich Salzer

**Corresponding authors:**

Bodo Grimbacher and Ulrich Salzer

Center for Chronic Immunodeficiency (CCI)

Medical Center – University of Freiburg

Faculty of Medicine

University of Freiburg

Germany

Email: bodo.grimbacher@uniklinik-freiburg.de/ulrich.salzer@uniklinik-freiburg.de

## Online Resource: Protocol for Elution and Determination of IgG Levels from Dried Blood Spots

1. A spot with a diameter of 6 mm was punched out centrally from each dried blood spot (DBS) card using a Harris Uni-Core 6.0 mm punch and a punching mat (GE Healthcare Life Sciences, Chalfont St Giles, UK). The punches and punching mat were cleaned with distilled water and 60% isopropyl alcohol between punching of spots from different individuals.
2. The punched spots were placed into 1.5 mL of low-protein-binding Eppendorf tubes, and 1000 μL of elution buffer (phosphate-buffered saline (PBS)/Tween 20 0.05%) was added.
3. Blood from each punched spot was eluted using a thermal mixer at 1000 rpm for 2 hours at room temperature, followed by incubation and mixing at 4°C and 1000 rpm for a further 17 hours. Subsequently, the eluate was equilibrated at room temperature and briefly spun down to collect the eluate at the bottom of the tube.
4. As an addition to the elution procedure described by Yel *et al*. [10], the punched spot and eluate were transferred to a 2 mL syringe (without a plunger) stacked in a 15 mL reaction tube, and the eluate was extracted by centrifugation at 2200 *g* for 10 minutes at room temperature.
5. For nephelometric analysis, eluates were diluted 1:4 with PBS/Tween 20 0.05%, and the diluted eluate was stored at 4°C overnight. Immunoglobulin G (IgG) concentration was determined by nephelometry using N antiserum against human IgG (Siemens Healthcare GmbH, Erlangen, Germany) and the settings for CSF IgG determination (no dilution) on an Atellica^®^ NEPH 360 System (Siemens Healthcare GmBH).
6. Separate standard curves and internal controls (Siemens Healthcare GmBH) were used according to the manufacturer’s instructions.
7. The amount of serum in a punched spot (diameter 6 mm; area 28.3 mm^2^ [calculated as πr^2^]) was calculated in a method adapted from Andersen *et al.* [9], given that a completely covered blood spot area (diameter 12.5 mm; area 122.7 mm^2^) contains approximately 50 μL of blood, that blood is distributed evenly on the Whatman card, and that 50% of whole blood is serum. The volume of serum in a punched spot was calculated to be 5.76 μL (calculated as [[area of 6mm spot ÷ area of whole DBS] x 50] ÷ 2). The volumes of smaller spots may be calculated using the method described by Hall *et al.* [19].
8. The concentration of IgG in serum was calculated by multiplying the nephelometric result with a correction factor of 694.4 (calculated as [elution volume × dilution factor] ÷ serum volume in punched spot), using 1000 μL of elution buffer and a dilution of 1:4.
